# The Dispensaries of North-West India

**Published:** 1899-03-18

**Authors:** 


### THE DISPENSARIES OF NORTH-WEST INDIA.

A diminished total of patients treated throughout the year in the hospitals would, at first sight, seem to suggest that the country in which this reduction had occurred must necessarily have increased in health. According to the annual returns of the dispensaries and charitable institutions of the North-West Provinces of India and Oudh, rather the opposite is the case. The total number of both indoor and outdoor patients treated during the year 1897 was 262,943 less than in the preceding year. The decrease was not attributable to a splendid bill of health, but was due in the first place to the famine, on account of which a large number of patients, who in ordinary years would have attended the dispensaries, were treated in poor houses and relief work hospitals, 726,929 persons in all. Another cause was the fear excited by plague preventive measures, which deterred many patients from attending a dispensary of any kind. They preferred to put up with the ills they had rather than fly to others that they knew not of. But though the natives have so keen a horror of the means which are employed to prevent the spread of the plague, the same sentiment does not extend to the prevention of fever. Over 311 lb. of quinine were distributed during the year, free of cost by order of the Government, to the poor of those districts which had suffered most severely from famine; and Rs.8,416 were spent in quinine, conchona, febrifuge and other remedies, which were freely given away through the agencies of *thanas*, *patwaris*, &c., and gladly accepted. Moreover, upwards of 404 lb. of quinine were issued for sale at post offices in pice packets, and were bought by those who required the drug. In looking over the hospital reports and remembering the days not very long past when the women of India were practically without medical treatment of any kind, except at the hands of superstitious old women who knew nothing, one cannot help being struck by the female hospitals and dispensaries which have arisen in nearly every important centre. A total of 46 institutions where women are treated, with 14 lady doctors (one a native), 31 hospital assistants, five matrons, 21 nurses, eight midwives, 37 compounders, and six dressers for one part of India alone is fairly satisfactory, though it has been noted that the female dispensary in Gonda was more than fully occupied—a sure proof that there is still a want of further accommodation in some parts.

